# Indoor Spatiotemporal Contact Analytics Using Landmark-Aided Pedestrian Dead Reckoning on Smartphones

**DOI:** 10.3390/s23010113

**Published:** 2022-12-22

**Authors:** Lulu Gao, Shin’ichi Konomi

**Affiliations:** 1 Graduate School of Information Science and Electrical Engineering, Kyushu University, Fukuoka 819-0395, Japan; 2 Faculty of Arts and Science, Kyushu University, Fukuoka 819-0395, Japan

**Keywords:** COVID-19, contact awareness, spatiotemporal analytics, indoor positioning, pedestrian dead reckoning, landmark identification

## Abstract

Due to the prevalence of COVID-19, providing safe environments and reducing the risks of virus exposure play pivotal roles in our daily lives. Contact tracing is a well-established and widely-used approach to track and suppress the spread of viruses. Most digital contact tracing systems can detect direct face-to-face contact based on estimated proximity, without quantifying the exposed virus concentration. In particular, they rarely allow for quantitative analysis of indirect environmental exposure due to virus survival time in the air and constant airborne transmission. In this work, we propose an indoor spatiotemporal contact awareness framework (iSTCA), which explicitly considers the self-containing quantitative contact analytics approach with spatiotemporal information to provide accurate awareness of the virus quanta concentration in different origins at various times. Smartphone-based pedestrian dead reckoning (PDR) is employed to precisely detect the locations and trajectories for distance estimation and time assessment without the need to deploy extra infrastructure. The PDR technique we employ calibrates the accumulative error by identifying spatial landmarks automatically. We utilized a custom deep learning model composed of bidirectional long short-term memory (Bi-LSTM) and multi-head convolutional neural networks (CNNs) for extracting the local correlation and long-term dependency to recognize landmarks. By considering the spatial distance and time difference in an integrated manner, we can quantify the virus quanta concentration of the entire indoor environment at any time with all contributed virus particles. We conducted an extensive experiment based on practical scenarios to evaluate the performance of the proposed system, showing that the average positioning error is reduced to less than 0.7 m with high confidence and demonstrating the validity of our system for the virus quanta concentration quantification involving virus movement in a complex indoor environment.

## 1. Introduction

The worldwide COVID-19 pandemic has brought about many changes in our daily lives and struck a devastating blow to the global economy. It is widely recognized that airborne transmission serves as the primary pathway for the spread of COVID-19 via expiratory droplets, especially in indoor environments [1]. During the viral outbreak, many people were infected due to exposure to virus droplets generated by human exhalation activities [2,3,4]. Some infected patients spread the virus unknowingly without properly being examined because there is an incubation period that varies for different mutations and asymptomatic patients who never experience apparent symptoms [5]. Reliable and efficient tracing and quarantining have become more important than ever to alert individuals to take actions to interrupt the transmission between people and further curb the spread of the disease. Contact tracing involves identifying, assessing, and managing people who are at risk of the infection, and tracking subsequent victims as recorded by the public health department [6]; contact tracing can be performed via manual or digital methods. Since the manual contact tracing is labor-intensive and time-consuming and may be incomplete and inaccurate due to forgetfulness; automatic digital contact tracing has been widely researched in recent years [7]. Usually, digital contact tracing applications are installed on portal devices, typically smartphones, to conveniently and intelligently realize tracing with the help of existing sensors based on various technologies, such as a global navigation satellite system (GNSS), Bluetooth, and Wi-Fi.

Contact tracing in indoor environments can complement the ones used in outdoor environments to enable comprehensive digital contact tracing. However, indoor contact tracing imposes unique technical challenges due to virus concentrations and unreliable GNSS signals in indoor environments [8]. The virus concentration, which plays a critical role in calculating the amount of a virus we are exposed to and further assesses the infection risk, should be explicitly considered in indoor contact tracing applications [3,9]. The quantitative infection risk for a susceptible person is significantly associated with the quantity of the pathogen inhaled in the surrounding ambient air, from the respiratory droplets exhaled by infected individuals [10]. Thus, inhaling a large amount of the virus in a short period, i.e., under the 15 min time mark, can greatly increase the infection risk, especially for so-called “superspreading events”, which invariably occur indoors [11]. Moreover, the majority of time has to be spent by people in indoor contexts with plenty of daily activities performed. However, GNSS-based approaches do not work well in indoor environments due to signal attenuation. Phone-to-phone pairing-based methods using Bluetooth low energy (BLE) work only for direct face-to-face contact tracing scenarios and are inapplicable to indirect virus exposure in ambient aerosols. The expelled pathogen-containing particles can remain active in the air for hours without sufficient sanitization, especially in indoor environments, constructing a significant fraction of the virus concentration [3]. Recently, vContact was proposed as a means to detect exposure to the virus with the consideration of asynchronous contacts by leveraging Wi-Fi networks, while the spatiotemporal dynamism in the virus concentration is not fully being considered [8].

Although the virus concentration will gradually decrease due to inactivation, deposition, and air purification after the virus-laden droplets are exhaled, the poor air exchange rate, superspreaders, and more virulent variants will keep it at a relatively high concentration for a long time in an indoor environment [9,12]. The viral particles are continuously ejected by infected people at different locations, relying on human movement. Moreover, due to the initial motion state and environmental airflow, these droplets maintain a ceaseless transmission before they are removed and meet somewhere (at some time), which leads to constant changes in the virus concentration within the control volume [13]. To accurately estimate the concentration, investigating the airborne transmission of these ejected particles is, thus, of fundamental importance in a closed environment because of the assemblage, in which human movement is implicitly involved to achieve the initial motion state of droplets [13]. The qualitatively location-specific assessment of the viral concentration is proposed with the dual use of computational fluid dynamic simulations and surrogate aerosol measurements for different real-world settings [14]. Moreover, the transmission of the virus brings about changes in the viral concentration of a specific location in an overall space, as well as the movements of people. Z. Li et al. analyzed the dispersion of cough-generated droplets in the wake of a walking person [4].

To be precisely aware of the amount of the virus one is exposed to and to detect both direct and indirect contacts, an indoor spatiotemporal contact awareness (iSTCA) framework is proposed. Since the virus concentration (at different times in the same area) is not the same because of the dispersion and diffusion of the virus and human movements, we employed a self-contained PDR technique to calculate the human trajectory with accuracy and further achieve the location and time of the expelled virus droplets for the quantitative measurement of the concentration at any time in different spots. Moreover, based on the acquired changing virus concentration and reliable trajectories, the exposure time and distance of both direct and indirect contacts can be derived via cross-examination to realize quantitative spatiotemporal contact awareness.

Our main contributions are as follows:

1. To accurately present the virus concentrations at different times, we established quantitative virus concentration changes in various areas of indoor environments at different times by infected individuals. The viral-laden droplets were continuously released during the expiratory activities, moving forward. During the movements of viral-loaded droplets exhaled by infectious individuals at different locations and times, the virus instances met in certain spots at certain times and contributed to the calculation of the concentration. Finally, the concentration of each virus instance was integrated.
2. We properly selected PDR for the acquisition of the trajectory to conduct contact awareness without requiring extra infrastructure or being affected by coverage limitations compared with other indoor positioning techniques.
3. We considered various landmarks to calibrate the accumulative error for trajectory achievement by using PDR. A custom deep neural network using bidirectional long short-term memory (Bi-LSTM) and multi-head convolutional neural networks (CNNs) with residual concatenations were designed and implemented to extract temporal information in forward and backward directions and spatial features at various resolutions from built-in sensor readings for landmark identification.
4. Additionally, we demonstrate the effectiveness of the proposed Bi-LSTM-CNN classification model for landmark identification through empirical experiments, as well as the performance of our proposed iSTCA system for quantitative spatiotemporal contact analytics.

The remainder of this paper is organized as follows. The related work about contact awareness and indoor localization techniques, including PDR, is reviewed in Section 2. Definitions and preliminaries about virus concentrations and different contact types are introduced in Section 3. Section 4 introduces the theoretical methodology and the architecture of the proposed iSTCA. The experimental methodology and results based on the collected datasets are presented in Section 5. Section 6 reveals the limitations of this work. Finally, we present the conclusion and future work in Section 7.

## 2. Related Work

Contact tracing is used to identify and track people who may have been exposed to a virus due to the prevalence of many infectious diseases in our society. To conduct contact tracing, it is necessary for the infected individuals to provide their visited locations and people whom they encountered based on the specific definitions of meetups for different diseases. Instead of interviews and questionnaires via traditional manual tracing, technology-aided contact tracing can track people at risk conveniently and intelligently. To reduce the spread of COVID-19 effectively, digital contact tracing, which generally depends on applications installed on smartphones, has been developed in both academia and industry, using various technologies, such as GNSS, Bluetooth, and Wi-Fi.

There are typically two approaches for encounter determinations, peer-to-peer proximity detection-based and geolocation-based. Peer-to-peer proximity can be estimated by the received signal strength (RSS) of wireless signals, such as Bluetooth and ultra-wideband (UWB), and the distance between two devices in geolocation-based approaches can be precisely derived from the cross-examination after obtaining the accurate location and trajectory with the help of localization techniques using various technologies, such as global positioning system (GPS), Wi-Fi, and PDR.

Some systems based on peer-to-peer proximity using Bluetooth or BLE have been implemented, and part of them are deployed by the governments of various countries, such as Australia (COVIDSafe), Singapore (Trace together), and the United Kingdom (NHS COVID-19 App) due to their ubiquitous embedding in mobile phones [15]. Among these systems, the most representative protocols are Blue Trace and ROBERT [11,16]. The data from Bluetooth device-to-device communications are stored and checked against the data uploaded by the infector. In Blue Trace, the health authority contacts individuals who had a high probability of virus exposure, whereas ROBERT users need to periodically probe the server for their infection risk scores. In addition, Google and Apple provide a broadly used toolkit based on Bluetooth, named Google and Apple Exposure Notification (GAEN), to facilitate a contact tracing system in Android and iOS and curb the spread of COVID-19 [17]. Despite some minor differences in implementation and efficiency, these schemes are all independently designed and very similar. When exposure is detected, the RSS in the communication data frame is utilized to estimate the distance between two devices and notify the user. However, it has been demonstrated that the signal strengths can only provide very rough estimations of the actual distances between devices, as they are affected by device orientation, shadowing, shading effects, and multipath losses in different environments [18,19]. Although it is difficult to measure the distances among users accurately by using Bluetooth and other technologies, the UWB radio technology has the capacity to measure distances at the accuracy level of a few centimeters, which is significantly bettering than Bluetooth [20]. The use of UWB, however, has some significant drawbacks, including the fact that UWB is not widely supported by mobile devices, requires extra infrastructure, and is not energy efficient, which makes UWB less useful in practice [21]. All of the above works that are based on calculated proximity using RSS do not consider the user’s specific physical location, resulting in unsatisfactory tracing results. Moreover, these approaches cannot be applied to the detection of temporal contact due to the dispersion and lifespan of the virus.

To achieve accurate geolocation in contact tracing, plenty of localization systems have been researched with the joint efforts of researchers and engineers in the past based on GNSS, cellular technology, radio frequency identification (RFID), and quick response (QR) code [7]. GNSS can be used for contact tracing as the exact position of a person can be located and it is available globally. Many countries, including Israel (HaMagen 2.0) and Cyprus (CovTracer), use GPS-based contact tracing approaches [15] as well. GNSS signals are usually weak in indoor environments due to the absence of the line of sight and the attenuation of satellite signals, as well as the noisiness of the environment. Many people may spend most of their time in indoor environments, which can result in limited contact coverage. It is difficult to detect contact based on cellular data due to the large coverage of cell towers and high location errors [8]. RFID was used to reveal the spread of infectious diseases and detect face-to-face contact in [22,23]. QR codes for contact tracing require users to check in at various venues by scanning the placed QR codes manually to record their locations and times, which are deployed in some countries, such as New Zealand (NZ COVID Tracer) [15]. However, special devices or codes have to be deployed at scale for data collection. Recently, some protocols were proposed for Wi-Fi-based contact tracing with the pre-installed Wi-Fi Access Point. WiFiTrace was proposed by proposed in [24]. WiFiTrace is a network-centric contact tracing approach with passive Wi-Fi sensing and without client-side involvement, in which the locations visited are reconstructed by network logs; graph-based model and graph algorithms are employed to efficiently perform contact tracing. Wi-Fi association logs were also investigated in [25] to infer the social intersections with coarse collocation behaviors. Li et al. utilized active Wi-Fi sensing for data collection; they leveraged signal processing approaches and similarity metrics to align and detect virus exposure with temporally indirect contact [8]. As the changes in virus concentrations over time (due to the transmission of aerosols and environmental factors) are not considered, their results are in relatively low spatiotemporal resolutions. The approach presented in [26] divides contact tracing into two separate parts, duration and distance of exposure. The duration is captured from the Wi-Fi network logs and the distance is calculated by the PDR positioning trajectory, calibrated by recognized landmarks with the help of a CNN, ensuring the performance of contact tracing. Although integration with the existing infrastructure is beneficial in mitigating the deployment costs, it may not fully satisfy the requirements of contact tracing with the high spatiotemporal resolution because of the absent coverage [27]. The trajectory obtained by the PDR technique, without requiring special infrastructure, can improve the coarse-grained duration and make it fine-grained. This can enable the development of a contact-tracing environment that considers the virus lifespan in detail.

One of the ultimate goals of contact awareness systems is to estimate the risk based on the recorded encounter data [28]. Moreover, with the exposure duration and distance obtained, the virus concentration is significant to determine the exposed viral load, which is closely associated with the infection risk [29]. Typically, the virus concentration in a given space depends on the total amount of viral load contained in the viable virus-laden droplets in the air and maintains a downward trend because of the self-inactivation and environmental factors. Researchers presented the qualitative location-specific assessment of viral concentration with the dual use of computational fluid dynamic simulations and surrogate aerosol measurements for different real-world settings [14]. The practical viral loads emitted by contagious subjects based on the viral loads in the mouth (or sputum) with various types of respiratory activities and activity levels are presented in [29]. Furthermore, to quantitatively shape the virus concentration in a targeted environment at different times, the constant viral load emission rate is adopted with the virus removal rate, including the air exchange rate, particle sediment, and viral inactivation rate in [30].

The aforementioned contact tracing research usually only considers the static virus concentration without considering the exposure to the environmental virus and dynamism in the virus concentration. Moreover, in contrast to the qualitative estimation of exposure risks that can be achieved in previous works, there is a lack of sufficient quantitative awareness about the concentrations of contracted viruses. Such awareness would be useful in our daily lives to protect ourselves from virus infections.

## 3. Definitions and Preliminaries

Virus-encapsulating secretions are continuously exhaled and aerosolized into airborne virus-laden particles with infectivity from daily expository activities. There is a great difference between the size and number of droplets expelled, depending on their origin locations in the respiratory tract [4]. The time and distances of these droplets traveling in indoor environments largely depend on the expiration air jet, particle weight, and ambient factors. The movements and the viral loads of virus-containing particles are directly associated with the virus concentrations in different regions. To quantitatively become aware of the exposure of the virus, the quanta concentration as a medical virus concentration indicator, virus airborne pattern, and various contact types are present.

### 3.1. Quanta Concentration

The viral loads of virus-containing droplets change after leaving the human expiratory tract with airborne transmission and a combination of environmental factors. In particular, the viral load emitted is expressed in terms of the quanta emission rate ($ER_{q}$, $\mathrm{quanta}\cdot h^{-1}$), in which a quantum is defined as the dose of airborne droplet nuclei that infect 63% of susceptible persons with exposure [30]. The quanta concentration in an indoor area at time $t,\;q\left(t\right)$ is measured by:

(1) $$q\left(t,ER_{q}\right)=N_{I}\cdot \frac{ER_{q}}{RR_{iv}\;\cdot \;V}+\left(q_{0}+N_{I}\cdot \frac{ER_{q}}{RR_{iv}}\right)\cdot \frac{e^{-RR_{iv}\cdot t}}{V}\left(\mathrm{quanta}\cdot m^{-3}\right)$$

where $ER_{q}$ is the quanta emission rate of the infector (measure in $\mathrm{quanta}\cdot h^{-1}$), $q_{0}$ is a constant declaring the initial number of quanta in the space, $V\;\left(m^{3}\right)$ is the target indoor volume, $N_{I}$ represents the number of infected individuals in the investigated volume, $RR_{iv}\;\left(h^{-1}\right)$ is the removal rate for the infectious virus in the considered spaces [30]. $RR_{iv}$ consists of three contributions, the air exchange rate (AER) via ventilation, the deposition on surface rate (k) caused by gravitational sedimentation and turbulent eddy impaction, and the viral inactivation rate (λ). The typical k is 0.24 $h^{-1}$ and the inactivation rate λ of viable COVID-19 particles in a typical indoor environment without sunlight is generally 0.63 $h^{-1}$, as indicated in [30,31].

The $ER_{q}$ is determined by the viral load in sputum, the volume of signal droplets, and the quantity of all expelled droplets per exhalation. Thus, the quanta concentration $ER_{q}$ is modeled as:

(2) $$ER_{q}=c_{v}\cdot c_{i}\cdot IR\cdot \int N_{d}\left(D\right)\cdot dV_{d}\left(D\right)\;\left(\mathrm{quanta}\cdot h^{-1}\right)$$

where $c_{v}$ represents the viral load in the sputum of the infector (RNA copies $\cdot {\mathrm{mL}}^{-1}$), IR is the inhalation/exhalation rate produced by the breathing rate and tidal volume, $N_{d}$ is the droplet concentrations in different expiratory activities of the infected person ($\mathrm{particles}\cdot {\mathrm{cm}}^{-3}$), $V_{d}$ is the volume of a single droplet (${\mathrm{cm}}^{3}$) with the function of particle diameters D, and $c_{i}$ is the conversion factor, presenting the ratio between one infectious quantum and the infectious dose expressed in the viral RNA copies [29]. There is a wide range of variations in the quanta emission estimation via Equation (2), depending on these and other factors, such as virus concentration in the mouth, activity level, and the type of coughing or exhaling. With light exercise and speaking, a quanta concentration of 142 ($\mathrm{quanta}\cdot h^{-1}$) can be obtained, which was widely adopted in many works [29].

### 3.2. Spatial–Temporal Contact

COVID-19 contained in expiratory droplets and expelled from the infector is transported and dispersed in the ambient airflow before finally being removed, inactivated, and inhaled by a susceptible. There are a number of factors that contribute to the droplet’s movement, such as the horizontally emitted velocity, the particle weight and the external environment. Occasionally, coughing and sneezing generate more particles with higher initial velocities ($11.7\;m\cdot s^{-1}$ for coughing) and virus quanta concentrations, while constantly performed breathing and speaking ($3.9\;m\cdot s^{-1}$ for speaking) produce fewer particles with relatively lower initial velocity and virus quanta concentrations [29]. Large droplets usually settle quickly in a few seconds or minutes owing to gravitational sedimentation and are evaporated into small nuclei in indoor environments, where the particle can disperse for a long distance in the vaporization process. Tiny particles, including ones that are evaporated and originally expelled, are trapped and carried continuously forward within a moist, warm, turbulent cloud of gas, with the help of airflow movement. To facilitate the calculation, the movement of each virus-laden droplet expelled at each moment is independent and divided into two stages, maintaining a uniform motion with the initial horizontal velocity (e.g., $3.9\;m\cdot s^{-1}$), being well-mixed within the moved space in the first phase (e.g., 1 s), and then instantaneously and evenly distributed in the overall considered space.

The contact in COVID-19 contact tracing is originally equivalent to direct face-to-face contact, while due to the transmission of the virus and survival time in the air, more cases of indirect contact have emerged [2]. Here, indirect contact mainly represents the asynchronous time contact, called temporal contact. Direct and indirect contacts are types of spatiotemporal contacts. If there is no time difference between two people, and only a spatial distance is presented, it is called spatial contact. Similarly, if there is no space difference between two people, and only a temporal distance is presented, it is called temporal contact. There are time and space gaps, a mixture of two single cases, called spatiotemporal contacts. Since both the time and space differences would decrease the virus quanta concentration, it is necessary to obtain the accurate value for the precise awareness of the virus quanta concentration.

## 4. Methodology

This work utilizes the trajectories, including the spatial position coordinates and time obtained by the PDR technique to quantitatively estimate the time-dependent changes in the virus quanta concentration derived from the movement and lifespan of the virus in various places of the considered indoor environment. The overview of the proposed scheme is systematically introduced in Section 4.1. In Section 4.2, we provided the data processing approaches utilized in PDR-based trajectory construction and the estimation of droplet exhalation. The PDR technique (with a calibration of the landmark recognized by a landmark identification model based on a residual Bi-LSTM and CNN structure) is discussed in Section 4.3. Further, the contact awareness model relying on the precisely constructed pedestrian trajectory is detailed in Section 4.4.

### 4.1. System Overview

**Figure 1:**
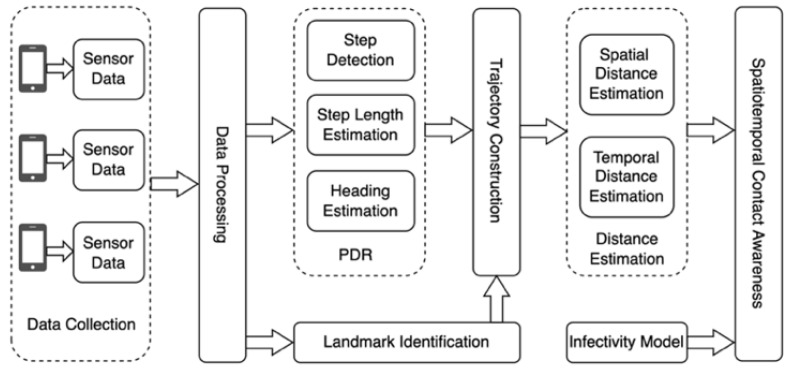
Overview of iSTCA.

An overview of the proposed iSTCA system is presented in Figure 1. More precisely, the data flow of various sensors for the analysis was primarily collected from the existing sensors in handhold smartphones, which record the changes in the environment and body motion. The signals need to be processed, including data filtering and scaling, to reduce the noise for a better state of motion estimation before training the landmark identification model and performing the PDR. The trajectory can be achieved based on the PDR technique and properly corrected with the assistance of the identified landmark distinguished by the trained landmark recognition model. The trajectory is defined as a set of points consisting of the time and position, $\left\{\left(t_{0},x_{0},y_{0}\right),\left(t_{1},x_{1},y_{1}\right),\ldots \left(t_{n},x_{n},y_{n}\right)\right\}$ where $\left(x_{i},y_{i}\right)$ represents the location coordinates and $t_{i}$ is the moment when the individual passes the location. The virus quanta concentrations in different spaces at various moments can be measured quantitatively to achieve sufficient awareness with the help of the estimated spatial distance, temporal distance, and infectivity model, as shown in Equation (1).

### 4.2. Data Preprocessing

**Data alignment.** The same sampling rate for data collection is set to 50 Hz due to the low frequency of human movements [32]. Although the constant rate is defined, the time interval between the recorded adjacent readings of each sensor is not always the same because of the observational error and random error, and it oscillates within a certain range in practice. To acquire the same number of samples for conveniently performing the subsequent procedures, we take the timestamp of the first data collected as the starting time to align the sensor readings at the same time interval with the help of data interpolation.

**Data interpolation.** During the practical data collection using smartphone sensors, some data points in the acquired dataset are lost due to malfunctioning; such data points are typically replaced by 0, NaN, or none [33]. To fill in the missing values, the data interpolation technique was developed, in which the new data point is estimated based on the known information. Linear interpolation, as the prevalent type of interpolation approach, was adopted in this paper, using linear polynomials to construct new data points [34]. Generally, the strategy for linear interpolation is to use a straight line to connect the known data points on either side of the unknown point and, thus, it is defined as the concatenation of linear interpolation between each pair of data points on a set of samples.

**Data filtering.** Due to the environmental noise and interference caused by the unconscious jittering of the human body, there are many undesirable components in the obtained signals that need to be dealt with [34]. This usually means removing some frequencies to suppress interfering signals and reduce the background noise. A low-pass filter is a type of electronic filter that attempts to pass low-frequency signals through the filter unchanged while reducing the amplitude of signals with a frequency above what is known as the cutoff frequency. A Butterworth low-pass filter with a cutoff frequency of 3 Hz is applied to denoise and smooth the raw signals.

**Data scaling.** The difference in the scale of each input variable increases the difficulty of the problem being modeled. If one of the features has a broad range of values, the objective functions of THE established model will be highly probably governed by the particular feature without normalization, suffering from poor performance during learning and sensitivity to input values and further resulting in a higher generalization error [35]. Therefore, the range of all data should be normalized so that each feature contributes approximately proportionately to the final result. Standardization makes the values of each feature in the data have zero means by subtracting from the mean in the numerator and unit variance, as shown in Equation (3):

(3) $$X_{i}^{'}=\frac{X_{i}-\mu }{\sigma }\;\left(i=1,\;2,\;3\cdots ,\;n\right)\;$$

where the $X_{i}^{'}$ is the standardized data, n represents the number of data channels, and μ and σ are the mean and standard deviations of the i-th channel of the samples [35]. This method is widely used for normalization in many machine learning algorithms and is also adopted in this work to normalize the range of data we obtained.

**Data segmentation.** A sensor-based landmark recognition model is typically fed with a short sequence of continuously recorded sensor readings since only a single data point cannot reflect the characteristics of landmarks. The sequence consists of all the channels of selected sensors. To preserve the temporal relationship between the acquired data points with the aligned times, we partition the multivariate time-series sensor signals into sequences or segments leveraging the sliding operation, which consists of 128 samples (corresponding to 2.56 s for the sampling frequency at 50 Hz) [34,36]. It is noteworthy that the length of the window is picked empirically to achieve the segments for all considered landmarks, in which the features of the landmarks can be precisely captured to promote the landmark identification model training [32,37].

### 4.3. PDR-Based Trajectory Construction Model

For the quantitative evaluation of the virus quanta concentration, the precise spatial distance and temporal distance between two individuals should be efficiently estimated. To reach this objective, a variety of indoor positioning techniques have been proposed for various scenarios. The widely studied fingerprinting-based method relies on the latest fingerprint database that needs to be precisely updated in time. In addition to the time-consuming and labor-intensive collection and re-establishment, the instability of RSS due to environmental uncertainties poses another challenge to the accuracy [38]. Moreover, coverage and distribution are also not satisfied in countries with poor ICT infrastructure [39]. Therefore, the self-contained PDR algorithm without extra requirements and coverage limitations is employed in this work, and its accuracy is improved by the identified landmark.

#### 4.3.1. PDR

Since PDR does not need additional equipment or a pre-survey, it has a wide range of potential applications for the indoor positioning of pedestrians. It relies on the inertial sensors extensively existing in mobile devices, e.g., smartphones, to acquire information about the user’s movements, which are then combined with the user’s previous location to estimate the present position and further achieve complete trajectory. The equation utilized for location estimation is as follows:

(4) $$\left\{\begin{array}{c} x_{t}=x_{t-1}+SL_{t}sin\theta_{t}\\ y_{t}=y_{t-1}+SL_{t}cos\theta_{t} \end{array}\right.\;$$

where $\left(x_{t},y_{t}\right)$ is the pedestrian position at time t, $SL_{t}$ is the step length, and $\theta_{t}$ details the heading direction of the pedestrian [40].

As mobile technology continues to evolve, a growing number of physical sensors are being installed in smartphones and, thus, various combinations of sensors can provide increasingly rich information, which makes PDR more feasible and accessible. A typical PDR consists of three main components: step detection, step-length estimation, and heading estimation [41].

**Step detection.** As the most popular method for accurate step detection, peak detection is employed in this paper, which relies on the repeating fluctuation patterns during human movement. Using the smartphone’s accelerometer to determine whether the pedestrian is stationary, or walking is straightforward as it directly reflects the moving acceleration. The magnitude of acceleration on three dimensions $\left(a_{x},a_{y},a_{z}\right)$ instead of the vertical part is employed as the input for peak findings to improve the accuracy, which can be expressed as:

(5) $$a=\sqrt{a_{x}^{2}+a_{y}^{2}+a_{z}^{2}}\;$$

where $a_{x},a_{y},a_{z}$ denote the three-axis accelerometer values in the smartphone [42]. A peak is detected when a is greater than the given threshold. To further enhance the performance, the low-pass filter is further applied to the magnitude to reduce the signal noise. Due to the acceleration jitter, the incumbently detected peak points need to be eliminated. Hence, an adaptive threshold technique of the maximum and minimum acceleration is adopted to fit different motion states with a time interval limitation between adjacent detected steps.

**Stride length estimation.** Various linear and nonlinear methods are proposed to estimate the step length, which varies from person to person because of different walking postures determined by various factors, including height, weight, and step frequency. Therefore, it is not easy to precisely construct the same step-length estimation model. Some researchers assume that the step length is a static value affected by the individual characteristics of different users. On the contrary, the empirical Weinberg model estimates the stride length according to the dynamic movement state, which is closer to reality [43]. The model is given by:

(6) $$SL=k\sqrt[{4}]{a_{max}-a_{min}}$$

where k is the dynamic value concerned with the acceleration of each step and $a_{max}\;,a_{min}$ are the maximum and minimum accelerations for each step [44].

**Heading estimation.** Heading information is a critical component for the entire PDR implementation, which seriously affects localization accuracy. To avoid the accumulative error in the direction estimation based on the gyroscope, and short-term direction disturbances based on the magnetometer, the combination of the gyroscope and magnetometer is typically adopted for heading estimation [42]. The current magnetometer heading signals, current gyroscope readings, and previously fused headings are weight-averaged to form the fused heading. The weighting factor is adaptive and is based on the magnetometer’s stability as well as the correlation between the magnetometer and the gyroscope [44]. As they are already fused in the rotation vector achieved from the rotation sensor in the smartphone, the heading change can be calculated by a rotation matrix transformed from the rotation vector [45]. The rotation vector is defined as: $\left[x,y,z,w\right]$, and the matrix is defined as $M,M\in R^{3\times 3}$. The heading direction on three dimensions can be evaluated by:

(7) $$M=\left[\begin{array}{ccc} M_{11} & M_{12} & M_{13}\\ M_{21} & M_{22} & M_{23}\\ M_{31} & M_{32} & M_{33} \end{array}\right]=\left[\begin{array}{ccc} 1-2y^{2}-2z^{2} & 2xy-2zw & 2xz+2yw\\ 2xy+2zw & 1-2x^{2}-2z^{2} & 2yz-2xw\\ 2xz-2yw & 2yz+2xw & 1-2x^{2}-2y^{2} \end{array}\right]$$

(8) $$\theta =\left[\begin{array}{c} arctan2\left(M_{12},\;M_{22}\right)\\ \arcsin\left(-M_{32}\right)\\ arctan2\left(-M_{31},M_{33}\right) \end{array}\right]=\left[\begin{array}{c} arctan2\left(2xy-2zw,\;1-2x^{2}-2z^{2}\right)\\ \arcsin\left(-2yz-2xw\right)\\ arctan2\left(2yw-2xz,\;1-2x^{2}-2y^{2}\right) \end{array}\right]$$

#### 4.3.2. Landmark Identification Model

Although PDR methods can estimate the location and trajectory of pedestrians, low-cost inertial sensors built into smartphones provide poor-quality measurements, resulting in accuracy degradation. Moreover, the cumulative error, including the heading estimation caused by the gyroscope and step-length estimation error caused by an accelerometer, could be produced in the long-term positioning using PDR, increasing the challenge of precise localization collection. Therefore, it is necessary to prepare the reference points with the correct positions known during the movement to reduce the accumulated errors when the user passes. Spatial contexts, such as landmarks, can be properly chosen to calibrate the localization error based on the inherent spatial information without additional deployment costs. Landmark is defined as a spatial point with salient features and semantic characteristics from its near environment in indoor positioning systems, such as corners, stairs, and elevators [27]. These features can be observed for identification in one or a combination of different sensors as people pass through the landmark. The locations of these landmarks are presented by geographical coordinates or the relationships with other locations/areas, where people perform specific and predictable activities. Changes in motion are reflected in sensor readings, and different motions present different patterns. The specific activities that people perform when passing landmarks are also reflected in at least one sensor. Using the data of one sensor or the combination of data from multiple sensors, the changing pattern of a specific activity can be identified, and then the landmark can be recognized [46]. The identified landmark can be used as an anchor point to correct the path we obtained and improve the performance of the calculated trajectory.

Landmark identification involves classifying the sequences of various sensor data recorded at regular intervals by sensing devices, usually smartphones, into a well-defined landmark, which has been extensively regarded as a problem of multivariate time series classification. To address this issue, it is critical to extract and learn the features comprehensively to determine the relationship between sensing information and movement patterns. In recent years, numerous features have been attained in many studies on certain raw signal statistical aspects, such as variance, mean, entropy, kurtosis, correlation coefficients, or frequency domains via the integration of cross-formal codings, such as signals with Fourier transform and wavelet transform [47]. Moreover, the special thresholds of different features for various kinds of landmark recognition are specifically analyzed. For instance, the threshold of angular velocity produced by a gyroscope is usually used to detect the corner landmark, the acceleration changes can recognize the stairs. The combinations of different thresholds of various sensors forming the decision tree can detect the standing motion state to further distinguish common landmarks, such as corners, stairs, and elevators [48,49]. However, despite high accuracy, the calculation, extraction, and selection of features of different sensors for various landmarks are heuristic (with professional knowledge and expertise of the specific domain), time-consuming, and laborious [47].

To facilitate feature engineering and improve performance, artificial neural networks based on deep learning techniques have been employed to conduct activity identification without hand-crafted extraction. Deep learning techniques have been applied in many fields to solve practical problems with remarkable performance, such as image processing, speech recognition, and natural language processing, to solve practical problems [50,51]. Many kinds of deep neural networks have been introduced and investigated to handle landmark identification based on the complexity and unsureness of human movements. Additionally, CNN and LSTM are widely adopted with high accuracy rate activity recognition among the applied networks. CNN is commonly separated into numerous learning stages, each of which consists of a mix of convolutional operation and nonlinear processing units, as follows:

(9) $$h^{k}=\sigma \left(\sum_{l\in L}g\left(x^{l},w^{k}\right)+b^{k}\right)$$

where $h^{k}$ reveals the latent representation of the k-th feature map of the current layer, σ is the activation function, g denotes the convolution operation, $x^{l}$ indicates the l-th feature map of the group of the feature maps L achieved from the upper layer, $w^{k}$ and $b^{k}$ express the weights matrix and the bias of the k-th feature map of the current layer, receptively [52]. In our model, the rectified linear units (ReLU) were employed as the activation functions to subsequently conduct the non-linear transformation to obtain the feature maps, denoted by:

(10) $$\sigma \left(x\right)=\max\left(0,\;x\right)$$

More importantly, the convolution operation in CNN can efficiently capture the local spatial correlation features by limiting the hidden unit’s receptive field to be local [53]. CNN considers each frame of sensor data as independent and extracts the features for these isolated portions of data without considering the temporal contexts beyond the boundaries of the frame. Due to the continuity of sensor data flow produced by the user’s behavior, local spatial correlations and temporally long-term connections are both important to identify the landmark [52]. LSTMs with learnable gates, which modulate the flow of information and control when to forget previous hidden states, as variants of vanilla recurrent neural networks (RNNs), allow the neural network to effectively extract the long-range dependencies of time-series sensor data [54]. The hidden state for the LSTM at time t is represented by:

(11) $$h_{t}=\sigma \left(w_{i,h}\cdot x_{t}+w_{h,h}\cdot h_{t-1}+b\right)$$

where $h_{t}$ and $h_{t-1}$ are the hidden state at time t and t−1, respectively, σ is the activation function, $w_{i,h}$ and $w_{h,h}$ are the weight matrices between the parts, and b symbolizes the hidden bias vector. The standard LSTM cells barely extract the features from the past movements, ignoring the future part. To comprehensively capture the information for landmark identification, the Bi-LSTM is applied to access the context in both the forward and backward directions [55].

**Figure 2:**
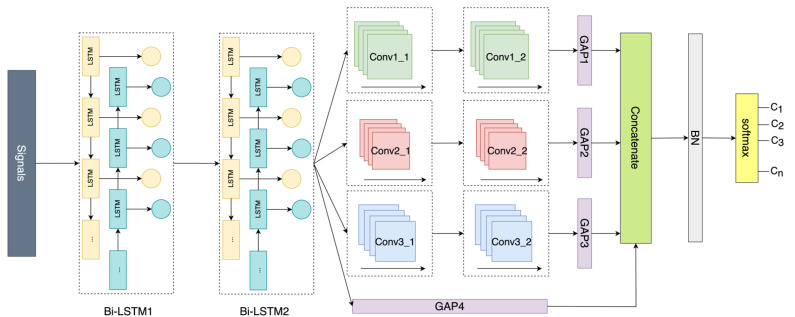
Architecture of the landmark identification model.

Therefore, both Bi-LSTM and CNN are involved in capturing the spatial and temporal features of signals for landmark identification. The architecture of the proposed landmark identification is shown in Figure 2. It performs the function of landmark recognition using the residual concatenation for classification, followed by Bi-LSTM and multi-head CNN. When preprocessed data segmentations of multiple sensors come, the inherent temporal relationship is extracted sequentially by two Bi-LSTM blocks that consist of a Bi-LSTM layer, a batch normalization (BN) layer, an activation layer, and a dropout layer. BN is a method used to improve training speed and accuracy with the mitigation of the internal covariate shift through normalization of the layer inputs by recentering and re-scaling [34]. Next, multi-head CNN blocks with varying kernels size are followed to learn the spatial features at various resolutions. Each convolutional block is made of four layers: a one-dimensional (1D) convolutional layer, a BN layer, an activation layer, and a dropout layer. To accommodate the three-dimensional input shape (samples, time steps, input channels) of the 1D convolutional layer, we retain the output of the hidden state in the Bi-LSTM layer. Then the acquired spatial and temporal features are combined, namely the concatenations of the outputs of the multi-head CNNs and Bi-LSTMs. To reduce the parameters and avoid overfitting, the global average pooling layer (GAP) with no parameter to optimize rather than the traditional fully connected layer is applied before combining the outputs [32]. Finally, the concatenated features are transmitted into a BN layer to re-normalize before being fed into a dense layer with a softmax classifier to generate the probability distribution over classes.

### 4.4. Contact Awareness with Trajectory

Exhalation and inhalation respiratory activities are constantly alternating (e.g., each breath consists of 2.5 s of continuous exhalation and 2.5 s of continuous inhalation), and droplets are continuously being released from the respiratory tract with a horizontal velocity during the process of exhalation with the same direction as the movement of the human. The particles exhaled at each moment will continue to move forward, starting from the user positions when they are expelled. The viral droplets exhaled from the infectious host are transported and dispersed into the ambient airflow before finally being inhaled by a susceptible person. Each exhalation lasts several seconds (e.g., 2.5 s), in which a long distance can be traveled for those who are in motion, and the initial position of droplets expelled cannot be accurately estimated in an indoor environment. Therefore, once complete, the exhalation period is divided into many short-term (e.g., 0.1 s) particle ejections. Because the interval is short, the continuous virus exhalation process can be converted into an instantaneous process, i.e., the virus is released instantly at the beginning of each interval. The virus-laden droplets expelled at different intervals maintain independent and identical motion patterns and the initial positions of the particles released in each interval can be regarded as the locations of the people at the initial moments. The virus-containing particles maintain a uniform motion of initially horizontal velocity (e.g., $3.9\;m\cdot s^{-1}$) in the first second and then instantaneously will mix in the overall considered space. Meanwhile, the droplets are evenly distributed within the moved space. In the first movement phase of the exhaled droplets in each interval, the virus moves in the same direction as the people travel, which is called forward transmission. As for the backward transmission, in general, the initial velocity of the virus is faster than the speed of movement and the speed of airflow, so in the first phase, very few virus particles move in the opposite direction.

The movements of all viral-loaded droplets exhaled by infectious people at different locations will meet somewhere at some time and contribute to the calculation of concentration. To precisely present the virus quanta concentrations, the transmissions of all virus particles per exhalation sources from different origins and in different states are assumed to follow the same patterns, in which the particles keep constant initial velocity in the first second and then will instantly mix in the overall space. The time it takes for the virus to move to the current point and the contribution to the virus quanta in the present are estimated with the help of spatial distance and velocity. Thus, the quanta concentration in an indoor area at time $t,\;q\left(t,\;ER_{q}\right)$ is measured by:

(12) $$q\left(t,\;ER_{q}\right)=\overset{i=N_{v}}{\underset{i}{\sum }}\left(\frac{ER_{q}^{i}}{RR_{iv}\;\cdot \;V\left(t^{i}\right)}\cdot \left(1+e^{-RR_{iv}\cdot t^{i}}\right)+\left(q_{0}\cdot \frac{e^{-RR_{iv}\cdot T}}{V}+q_{0}^{i}\cdot \frac{e^{-RR_{iv}\cdot t^{i}}}{V}\right)\right)$$

where $RR_{iv}$ is the virus removal rate of the target space, $N_{v}$ represents the virus generated in different places at different moments, $ER_{q}^{i}$ is the of the quanta emission rate of the infector at which the virus (i-th) is expelled, T is the time difference from the start of the experiment to present, $t^{i}$ is the time difference between the current time and the originating time of the virus (i-th), $V\left(t^{i}\right)$ is the volume of the space that the i-th virus had passed since it was expelled to the present, $q_{0}$ is the environmental virus quanta number, $q_{0}^{i}$ is the virus exhaled by the infector that has evenly spread to the overall investigated space with the volume of V. Exhaled virus particles eventually become the environmentally well-mixed virus quanta, while different initial states induce different decays.

### 4.5. Spatiotemporal Contact Awareness

The algorithm of the proposed iSTCA with the landmark-calibrated PDR technology based on a smartphone is detailed in Algorithm 1. The detailed procedures are as follows,

Firstly, the raw signals are acquired via the developed collection application and preprocessed to create the dataset for the landmark identification model training by utilizing the data preprocessing method introduced in Section 4.2.

**Algorithm 1** Indoor spatiotemporal contact awareness algorithmInput:raw sensor signals of infector’s smartphone,
trained landmark identification model $M_{lm}$

target time T

target position P

Infectivity model $M_{I}$
Output:quantitative virus quanta concentration in P at T.1.time interval initialized to τ, 2.quanta concentration in P at T ($q_{P}^{T}$) initialized to 0,3.construct the processed signals D,4.achieve the trajectories S from D, landmark-calibrated via $M_{lm}$,5.establish the initial state set $\left\{Q_{0}^{i}\right\}$ of all viruses expelled at different intervals, where $Q_{0}^{i}\leftarrow \left(t_{0}^{i},\;V_{0}^{i},\;q_{0}^{i}\right)$, i represent the number of time intervals,6.for each $Q_{0}^{i}$ do:7.for $j\;\mathrm{in}\;0,1,2,\ldots |\frac{T-t_{0}^{i}}{\tau }|$ do:8.achieve the $Q_{j}^{i}\leftarrow \;\left(t_{j}^{i},\;V_{j}^{i},\;q_{j}^{i}\right)$ based on movement pattern ($M_{I}$) itself9.if $P\;in\;V_{j}^{i}$ then:10.update $q_{P}^{T}$, $q_{P}^{T}\leftarrow q_{P}^{T}+q_{j}^{i}$
11.end if12.end for13.end for14.return $q_{P}^{T}$


Secondly, the landmark recognition model designed in Section 4.3.2 would be trained and stored based on the dataset generated in the first step to further the PDR algorithm.

Thirdly, the target trajectory S is constructed by performing the landmark-calibrated PDR technique, including step detection, stride length estimation, heading determination, and landmark identification.

Fourthly, we obtain the initial state set $\left\{Q_{0}^{i}\right\}$ of the expelled particles in the i-th $\left(i=1,2,3\ldots \right)$ short-term period with the help of the calculated human movement trajectory S and the preset viral particle ejection interval τ. $Q_{0}^{i}$ defines the state of all i-th emitted particles in interval τ and consists of three parts t,V,q, where t represents the elapsed time after being exhaled, V represents the spread coverage of droplets due to airborne dispersion, and q represents the quanta concentration.

Fifthly, the state set $\left\{Q_{j}^{i}\right\}$ at the j-th interval for any $Q_{0}^{i}$ after being expelled is acquired by employing the defined movement pattern of the considered particles.

Finally, the virus quanta concentration $q_{P}^{T}$ in the target position P at the target time T is reached. The virus quanta concentration presented within P at T by particles expelled in the various intervals is summed to estimate $q_{P}^{T}$. Moreover, the virus quanta concentrations presented in different locations at various times can be further evaluated.

## 5. Experiments

In this section, we evaluate the performances of the proposed methods through experiments with the dataset we collected in a university building. We introduce the experimental scenario and data collection in Section 5.1 and the results are presented in Section 5.2; we analyzed the performances related to the landmark identification, PDR, and virus quanta concentration.

### 5.1. Experimental Scenario and Data Acquisition

**Figure 3:**
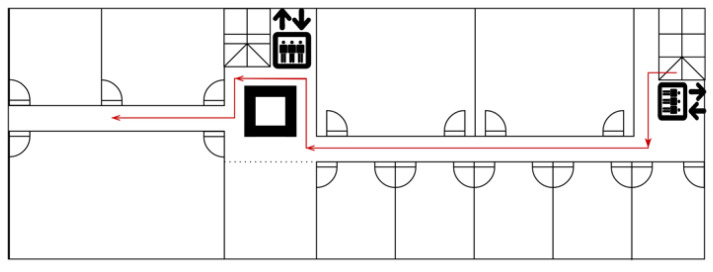
The floor plan of our experiment.

We collected our experimental data on the fourth floor of the Center-Zone-1 building of Kyushu University’s Ito campus. We assume that there is no exchange of virus particles with the room space. Figure 3 shows the floor plan of the experimental area. Based on the practical scenario, the Manhattan distance is applied to measure the virus movement. Since the width (measured as 2 m) and height (assumed to be 3 m based on the practical scenario) of the hallway are generally the same, the volume of the virus coverage can be determined by the virus movement distance for the calculation of the virus quanta concentration. When the virus encounters a corner, its direction changes, leading to a shift in the virus quanta concentration to varying degrees. For a corner with two branches, the concentration is assumed to decrease by half due to the inertia effect while these viral particles continue the forward transmission. If it is a corner with three or more branches, we assume that the virus quanta would be distributed evenly in all other directions.

In data collection, five recruited participants held Pixel 4a smartphones with the required sensors integrated (e.g., accelerometer, gyroscope, and rotation sensor) and an Android application installed. The application can periodically read and store the readings of 11 channels (3 for the accelerometer, 3 for the gyroscope, and 5 for the rotation sensor) as the user walks along the prescribed routes at a normal speed in the experimental area. Moreover, participants are required to hold their smartphones at chest level, which is a reasonable position where participants can record extra information to facilitate data processing. Indeed, it is recommended that they record the timestamp and the identification of the passing landmark to construct the dataset for the landmark identification model training.

### 5.2. Analysis and Discussion

#### 5.2.1. Landmark Identification

The proposed landmark recognition model was extensively evaluated by a series of experiments and implemented using the Keras framework with the TensorFlow backend to minimize the cross-entropy loss. The model was performed using the collected data with 3863 samples. The dataset was divided into training (70%) and testing (30%) sets, randomly, without overlapping. There were a total of 11 landmarks, including 7 corners, 2 stairs, and 2 elevators.

Table 1 details the network configuration considered in our study. Since there were many combinations of parameters, to reduce the selection space, we let all of the Bi-LSTM neurons share the same value, with the 1D convolution filter and kernel sizes accessing the same setting, respectively. To achieve stable performances of different model settings, a grid search with the 10-fold cross-validation method was adopted. It worked through all of the combinations of parameters to find the best settings. It should be noted that the following study uses the bold value for each parameter when it is not otherwise specified.

**Figure 4:**
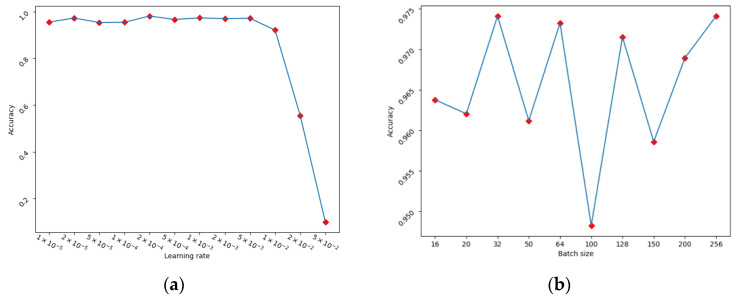
Model accuracy on various learning rates (**a**) and batch sizes (**b**), shown as the red square.

Moreover, the model configuration (the Adam optimization algorithm) was selected as the optimizer during the gradient descent. Other training hyperparameters were also evaluated and their recognition accuracies are presented in Figure 4. More specifically, the experiment was conducted with the learning rates of 0.00001, 0.00002, 0.00005, 0.0001, 0.0002, 0.0005, 0.001, 0.002, 0.005, 0.01, 0.02, and 0.05, as presented in Figure 4a. The mini-batch size was tested with 16, 20, 32, 50, 64, 100, 128, 150, 200, and 256, as shown in Figure 4b. The model configured as Table 1 achieved the highest identification accuracy of 98.4% when the learning rate was 0.0002 and the mini-batch size was 256. Additionally, early stopping criteria and a learning rate reduction strategy were applied during the model training process in order to reduce the issue of over-fitting and to improve the model performance. The learning rate decreased with a factor of 0.5 when the accuracy was not improved for 10 epochs and the training ended if the accuracy without enhancement on the validation was set after 15 iterations. Detailed training hyperparameter settings are revealed in Table 2.

**Figure 5:**
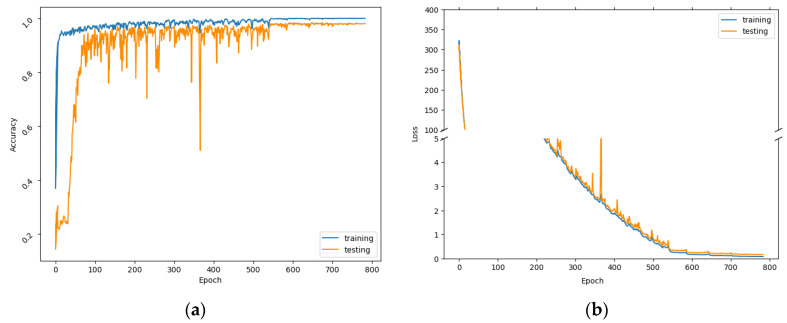
Accuracy (**a**) and loss (**b**) curves of the model on the selected parameters.

**Figure 6:**
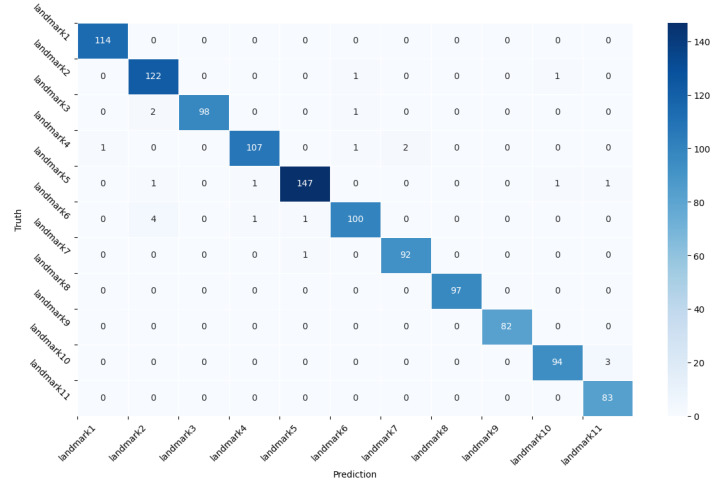
Confusion matrix for landmark identification.

Following the considered model configuration and optimal training hyperparameters, the accuracy curve and loss curve of the training and testing processes are illustrated in Figure 5a,b. The recognition results on 11 selected landmarks of the experiment-conducted floor are presented by the confusion matrix in Figure 6.

Moreover, to evaluate the proposed network more comprehensively, further comparisons were conducted on other deep neural networks (CNN, LSTM, and LSTM-CNN without residual connections) with the same depth and training hyperparameters as shown in Table 2. Table 3 presents the obtained experimental results of accuracy, precision, recall, and F1-score using different networks. It can be seen that the proposed Bi-LSTM-CNN network achieved the highest performance in all four metrics thanks to the elaborately extracted spatial and temporal features. Therefore, the effectiveness of the proposed Bi-LSTM-CNN classification model for the landmark identification task is demonstrated with the experimental evaluation.

#### 5.2.2. Trajectory Tracing

**Figure 7:**
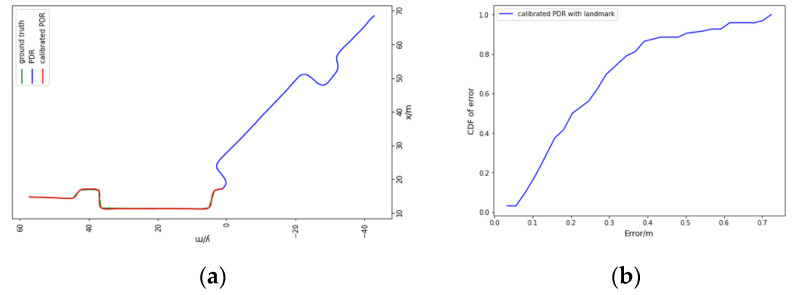
The performance (**a**) and the accumulative error distribution (**b**) of the proposed landmark-calibrated PDR.

The path shown as the red line in Figure 3 is designed to evaluate the performance of PDR with landmark calibration and the results are presented in Figure 7a. To quantitatively evaluate the positioning accuracy, we show the accumulative error distribution in Figure 7b. It can be seen from the left figure that the original PDR has an increasing error due to the initial wrong direction, although the information of many short segments can be described relatively accurately. Due to the significant error in the heading estimation without the landmark correction, the cumulative error distribution is not displayed in the right picture. The performance of PDR with the landmark calibration is well examined, nearly 80% of the positioning errors are less than 0.4 m, and the error probability within 0.7 m is higher than 90%. From the conducted experiments, the performance of the PDR-fused landmark calibration was evaluated with a lower positioning error, as compared to the PDR without calibration.

#### 5.2.3. Virus Quanta Concentration

As mentioned above, we regard all virus particles exhaled every 0.1 s during exhalation as virus instances. There will be many virus instances expelled during the entire movement of an infector. During the transmission of each instance, a uniform motion with a velocity of 3.9 ($m\cdot s^{-1}$) is maintained in the first second after exhalation, and the virus quanta are evenly distributed in the space that is passed by. The initial number of quanta ($q_{0}=0$), the virus quanta emission rate ($ER_{q}$), and the removal rate of infectious viral-laden particles ($RR_{iv})$ are 142 and 1.37, respectively, and remain the same within the experiment [29,30].

**Figure 8:**
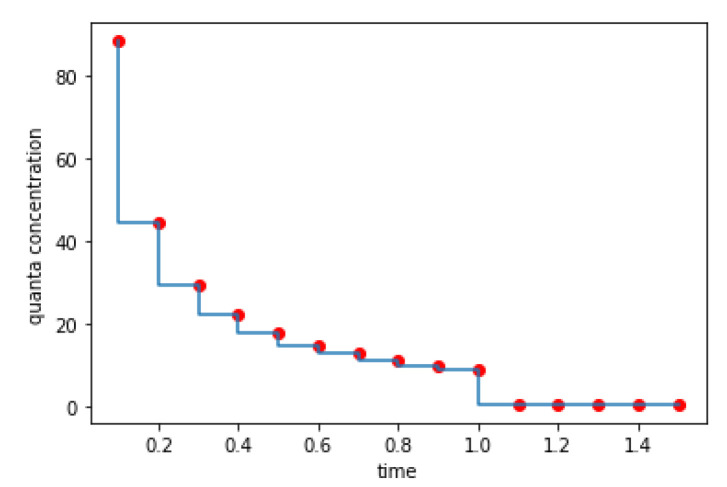
Quanta concentration of viral particles changes over time (first 1.5 s) after being released. Red points represent the instantaneous concentration at the end of each shorter interval.

The virus-laden particles released in each interval follow the same moving pattern, leading to the same trend in the change of the quanta concentration. We chose the instantaneous concentration at the end of each shorter interval with a length of 0.1 s to represent the concentrations at all times during the entire interval, as presented in Figure 8. As can be seen in Figure 8, the overall change in concentration presents an exponentially decreasing trend, from above 88 in the first interval (0~0.1 s) to close to 0 one second later. The sharp decrease one second later is because of an instantaneous expansion of the viral aerosol coverage to the entire considered space.

**Figure 9:**
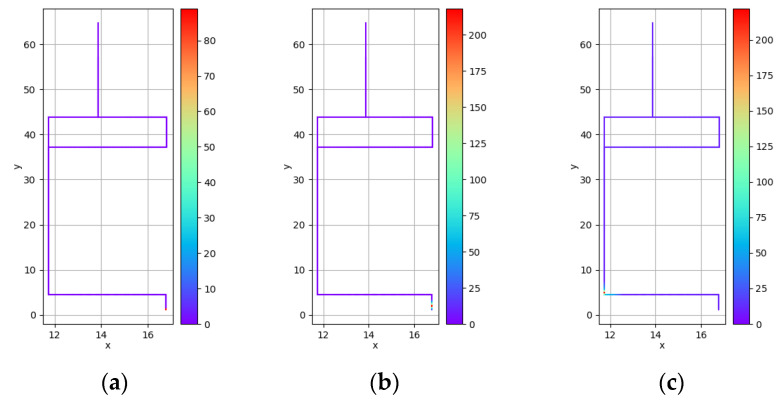
Indoor virus quanta concentrations at 0 s (**a**), 0.5 s (**b**), and 5 s (**c**), respectively, from the start of the movement.

The time when people started moving can be seen at time 0 of the experiment. Figure 9 presents the virus concentration in the current environment at the time of 0 s, 0.5 s, and 5 s from left to the right (using lines to represent the considered corridor spaces). Among them, at t = 0 s, only the virus concentration near the point start can be seen to exceed 80, while most of the other parts are not covered by viral particles. At t = 0.5 s, under the combined movements of virus droplets and humans, the relatively high quanta concentrations covered more. In addition, after another 0.5 s, the particles initially expelled at t = 0 s will spread to the overall space. At t = 5 s, the area with higher quanta concentration gradually moves forward with the movement of people. Moreover, due to the accumulated particles that diffuse into the entire environment, the quanta concentration in the overall space is increased, gradually reaching a non-negligible level compared with the concentration of the newly expelled virus instance.

## 6. Limitations

Although the proposed iSTCA system realizes quantitative representation for exposed virus concentrations with the help of the landmark-calibrated PDR technique, there are some challenges that need to be overcome. First of all, there are some strict restrictions in the data acquisition process. The participants are required to hold the smartphone, specifically the Pixel 4a, at chest level. As a result, except for the diversity of users considered, other factors that affect the motion sensor readings are not seriously taken into account, such as the mobile device heterogeneity (e.g., different types or various vendors) and the device’s status variation (e.g., putting in a pocket or handbag). In addition, a large amount of power of the smartphone is consumed during the indoor positioning process, resulting in the smartphone being overheated.

## 7. Conclusions and Future Work

Technology-assisted virus exposure tracking approaches are increasingly being adopted to mitigate and tame the epidemic. In view of the complexity of quantifying virus exposure due to human movement and airborne dispersion of virus particles, we propose iSTCA, a self-containing contact awareness approach that exploits PDR-based techniques. Quantitative information support directly concerned with risk assessment is provided for self-protection and epidemic control. More precisely, to reduce and calibrate the accumulative errors of trajectories based on landmarks, we apply Bi-LSTM and multi-head CNN with residual concatenation to long-term dependency in forward and backward directions and extract local correlations at various resolutions for landmark identification. The proposed method exploits the trajectories of people with viral-laden droplets exhaled and the transmission and attenuation of viruses in the air to quantify the virus quanta concentration in an indoor environment via spatiotemporal analytics for prevention and sanitization. In future work, we will continue studying the landmark identification model with different devices and various attitudes of the device and conduct further research on the exploration of other advanced deep neural networks and fusion algorithms. We will consider employing wearable devices, such as smartwatches and smart bands, to replace mobile phones for power saving in indoor positioning. Moreover, we plan to apply the proposed techniques for the development of services in developing communities without reliable digital infrastructure.

## Figures and Tables

**Table 1:** Landmark identification neural network configuration.

Layers	Parameter	Value
Input	shape	(None, 128, 11)
Bi-LSTM1	neurons	32, 64, **128**, 256
Bi-LSTM2	neurons	32, 64, **128**, 256
Conv1_1	kernel size	**3**, 5, 9, 11
	filters	32, 64, **128**
	stride	1
Conv2_1	kernel size	3, **5**, 9, 11
	filters	32, 64, **128**
	stride	1
Conv3_1	kernel size	3, 5, **9**, 11
	filters	32, 64, **128**
	stride	1
Conv1_2, Conv2_2, Conv3_2	kernel size	**3**, 5, 9, 11
	filters	32, **64**, 128
	stride	1
Dropout	drop rate	0.2, **0.3**, 0.5, 0.8

**Table 2:** Training hyperparameters.

Hyperparameters	Value
Optimizer	Adam
Activation function	ReLU
Batch size	256
Learning rate	0.0002
Epochs	800

**Table 3:** Landmark identification performances of different models in the collected dataset.

Method	Accuracy	Precision	Recall	F1-Score
CNN	0.9327	0.9404	0.9116	0.9258
LSTM	0.9637	0.9636	0.9600	0.9618
LSTM-CNN	0.9706	0.9750	0.9705	0.9727
Bi-LSTM-CNN (our)	0.9836	0.9849	0.9850	0.9849

## Data Availability

The data presented in this study are available upon request from the corresponding author. The data are not publicly available due to privacy reasons.

## References

[1] Shah Y., Kurelek J.W., Peterson S.D., Yarusevych S. (2021). Experimental Investigation of Indoor Aerosol Dispersion and Accumulation in the Context of COVID-19: Effects of Masks and Ventilation. Phys. Fluids.

[2] Marzoli F., Bortolami A., Pezzuto A., Mazzetto E., Piro R., Terregino C., Bonfante F., Belluco S. (2021). A Systematic Review of Human Coronaviruses Survival on Environmental Surfaces. Sci. Total Environ..

[3] Wang C.C., Prather K.A., Sznitman J., Jimenez J.L., Lakdawala S.S., Tufekci Z., Marr L.C. (2021). Airborne Transmission of Respiratory Viruses. Science.

[4] Li Z., Wang H., Zhang X., Wu T., Yang X. (2020). Effects of Space Sizes on the Dispersion of Cough-Generated Droplets from a Walking Person. Phys. Fluids.

[5] Ferretti L., Wymant C., Kendall M., Zhao L., Nurtay A., Abeler-Dörner L., Parker M., Bonsall D., Fraser C. (2020). Quantifying SARS-CoV-2 Transmission Suggests Epidemic Control with Digital Contact Tracing. Science.

[6] Alo U.R., Nkwo F.O., Nweke H.F., Achi I.I., Okemiri H.A. (2021). Non-Pharmaceutical Interventions against COVID-19 Pandemic: Review of Contact Tracing and Social Distancing Technologies, Protocols, Apps, Security and Open Research Directions. Sensors.

[7] Nguyen T.D., Miettinen M., Dmitrienko A., Sadeghi A.-R., Visconti I. (2022). Digital Contact Tracing Solutions: Promises, Pitfalls and Challenges. arXiv.

[8] Li G., Hu S., Zhong S., Tsui W.L., Chan S.-H.G. (2022). VContact: Private WiFi-Based IoT Contact Tracing with Virus Lifespan. IEEE Internet Things J..

[9] Faust J.S., Du C., Liang C., Mayes K.D., Renton B., Panthagani K., Krumholz H.M. (2022). Excess Mortality in Massachusetts During the Delta and Omicron Waves of COVID-19. JAMA.

[10] Bazant M.Z., Bush J.W.M. (2021). A Guideline to Limit Indoor Airborne Transmission of COVID-19. Proc. Natl. Acad. Sci. USA.

[11] Castelluccia C., Bielova N., Boutet A., Cunche M., Lauradoux C., Métayer D.L., Roca V. ROBERT: ROBust and Privacy-PresERving Proximity Tracing. https://hal.inria.fr/hal-02611265/document.

[12] Brüssow H. (2022). COVID-19: Omicron—The Latest, the Least Virulent, but Probably Not the Last Variant of Concern of SARS-CoV-2. Microb. Biotechnol..

[13] Wang J., Dalla Barba F., Roccon A., Sardina G., Soldati A., Picano F. (2022). Modelling the Direct Virus Exposure Risk Associated with Respiratory Events. J. R. Soc. Interface.

[14] Ooi C.C., Suwardi A., Ou Yang Z.L., Xu G., Tan C.K.I., Daniel D., Li H., Ge Z., Leong F.Y., Marimuthu K. (2021). Risk Assessment of Airborne COVID-19 Exposure in Social Settings. Phys. Fluids.

[15] Shahroz M., Ahmad F., Younis M.S., Ahmad N., Kamel Boulos M.N., Vinuesa R., Qadir J. (2021). COVID-19 Digital Contact Tracing Applications and Techniques: A Review Post Initial Deployments. Transp. Eng..

[16] Bay J., Kek J., Tan A., Hau C.S., Yongquan L., Tan J., Quy T.A. (2020). BlueTrace: A Privacy-Preserving Protocol for Community-Driven Contact Tracing across Borders.

[17] Leith D.J., Farrell S. GAEN Due Diligence: Verifying the Google/Apple COVID Exposure Notification API. Proceedings of the CoronaDef21, NDSS ‘21.

[18] Leith D.J., Farrell S. (2020). Measurement-Based Evaluation of Google/Apple Exposure Notification API for Proximity Detection in a Light-Rail Tram. PLoS ONE.

[19] Leith D.J., Farrell S. (2021). Measurement-Based Evaluation of Google/Apple Exposure Notification API for Proximity Detection in a Commuter Bus. PLoS ONE.

[20] Istomin T., Leoni E., Molteni D., Murphy A.L., Picco G.P., Griva M. (2022). Janus: Dual-Radio Accurate and Energy-Efficient Proximity Detection. Proc. ACM Interact. Mob. Wearable Ubiquitous Technol..

[21] Biri A., Jackson N., Thiele L., Pannuto P., Dutta P. SociTrack: Infrastructure-Free Interaction Tracking through Mobile Sensor Networks. Proceedings of the 26th Annual International Conference on Mobile Computing and Networking.

[22] Salathé M., Kazandjieva M., Lee J.W., Levis P., Feldman M.W., Jones J.H. (2010). A High-Resolution Human Contact Network for Infectious Disease Transmission. Proc. Natl. Acad. Sci. USA.

[23] Isella L., Romano M., Barrat A., Cattuto C., Colizza V., den Broeck W.V., Gesualdo F., Pandolfi E., Ravà L., Rizzo C. (2011). Close Encounters in a Pediatric Ward: Measuring Face-to-Face Proximity and Mixing Patterns with Wearable Sensors. PLoS ONE.

[24] Trivedi A., Zakaria C., Balan R., Becker A., Corey G., Shenoy P. (2021). WiFiTrace: Network-Based Contact Tracing for Infectious Diseases Using Passive WiFi Sensing. Proc. ACM Interact. Mob. Wearable Ubiquitous Technol..

[25] Swain V.D., Kwon H., Sargolzaei S., Saket B., Morshed M.B., Tran K., Patel D., Tian Y., Philipose J., Cui Y. (2020). Leveraging WiFi Network Logs to Infer Student Collocation and Its Relationship with Academic Performance. arXiv.

[26] Tu P., Li J., Wang H., Wang K., Yuan Y. (2021). Epidemic Contact Tracing with Campus WiFi Network and Smartphone-Based Pedestrian Dead Reckoning. IEEE Sens. J..

[27] Gao L., Konomi S. (2022). Mapless Indoor Navigation Based on Landmarks. Proceedings of the Distributed, Ambient and Pervasive Interactions. Smart Living, Learning, Well-Being and Health, Art and Creativity: 10th International Conference, DAPI 2022, Held as Part of the 24th HCI International Conference, HCII 2022, Virtual Event, 26 June–1 July 2022, Proceedings, Part II.

[28] Kindt P.H., Chakraborty T., Chakraborty S. (2021). How Reliable Is Smartphone-Based Electronic Contact Tracing for COVID-19?. Commun. ACM.

[29] Shen J., Kong M., Dong B., Birnkrant M.J., Zhang J. (2021). Airborne Transmission of SARS-CoV-2 in Indoor Environments: A Comprehensive Review. Sci. Technol. Built Environ..

[30] Buonanno G., Stabile L., Morawska L. (2020). Estimation of Airborne Viral Emission: Quanta Emission Rate of SARS-CoV-2 for Infection Risk Assessment. Environ. Int.

[31] van Doremalen N., Bushmaker T., Morris D.H., Holbrook M.G., Gamble A., Williamson B.N., Tamin A., Harcourt J.L., Thornburg N.J., Gerber S.I. (2020). Aerosol and Surface Stability of SARS-CoV-2 as Compared with SARS-CoV-1. N. Engl. J. Med..

[32] Tong L., Ma H., Lin Q., He J., Peng L. (2022). A Novel Deep Learning Bi-GRU-I Model for Real-Time Human Activity Recognition Using Inertial Sensors. IEEE Sens. J..

[33] Khatun M.A., Yousuf M.A., Ahmed S., Uddin M.Z., Alyami S.A., Al-Ashhab S., Akhdar H.F., Khan A., Azad A., Moni M.A. (2022). Deep CNN-LSTM with Self-Attention Model for Human Activity Recognition Using Wearable Sensor. IEEE J. Transl. Eng. Health Med..

[34] Xia K., Huang J., Wang H. (2020). LSTM-CNN Architecture for Human Activity Recognition. IEEE Access.

[35] Demrozi F., Turetta C., Pravadelli G. (2021). B-HAR: An Open-Source Baseline Framework for in Depth Study of Human Activity Recognition Datasets and Workflows. arXiv.

[36] Gu F., Khoshelham K., Valaee S., Shang J., Zhang R. (2018). Locomotion Activity Recognition Using Stacked Denoising Autoencoders. IEEE Internet Things J..

[37] Zhao Y., Yang R., Chevalier G., Xu X., Zhang Z. (2018). Deep Residual Bidir-LSTM for Human Activity Recognition Using Wearable Sensors. Math. Probl. Eng..

[38] Subedi S., Pyun J.-Y. (2020). A Survey of Smartphone-Based Indoor Positioning System Using RF-Based Wireless Technologies. Sensors.

[39] Konomi S., Gao L., Mushi D. (2020). An Intelligent Platform for Offline Learners Based on Model-Driven Crowdsensing Over Intermittent Networks. Proceedings of the Cross-Cultural Design. Applications in Health, Learning, Communication, and Creativity: 12th International Conference, CCD 2020, Held as Part of the 22nd HCI International Conference, HCII 2020, Copenhagen, Denmark, 19–24 July 2020, Proceedings, Part II.

[40] Li W., Chen R., Yu Y., Wu Y., Zhou H. (2021). Pedestrian Dead Reckoning with Novel Heading Estimation under Magnetic Interference and Multiple Smartphone Postures. Measurement.

[41] Liu T., Zhang X., Li Q., Fang Z. (2019). Modeling of Structure Landmark for Indoor Pedestrian Localization. IEEE Access.

[42] Yao H., Shu H., Sun H., Mousa B.G., Jiao Z., Suo Y. (2020). An Integrity Monitoring Algorithm for WiFi/PDR/Smartphone-Integrated Indoor Positioning System Based on Unscented Kalman Filter. EURASIP J. Wirel. Commun. Netw..

[43] Weinberg H. (2002). Using the ADXL202 in Pedometer and Personal Navigation Applications. Analog Devices 602 Appl. Note.

[44] De Cock C., Joseph W., Martens L., Trogh J., Plets D. (2021). Multi-Floor Indoor Pedestrian Dead Reckoning with a Backtracking Particle Filter and Viterbi-Based Floor Number Detection. Sensors.

[45] Yoon J., Kim S. (2022). Practical and Accurate Indoor Localization System Using Deep Learning. Sensors.

[46] Gu F., Valaee S., Khoshelham K., Shang J., Zhang R. (2020). Landmark Graph-Based Indoor Localization. IEEE Internet Things J..

[47] Nafea O., Abdul W., Muhammad G., Alsulaiman M. (2021). Sensor-Based Human Activity Recognition with Spatio-Temporal Deep Learning. Sensors.

[48] Zhou B., Li Q., Mao Q., Tu W., Zhang X., Chen L. (2015). ALIMC: Activity Landmark-Based Indoor Mapping via Crowdsourcing. IEEE Trans. Intell. Transp. Syst..

[49] Wang X., Jiang M., Guo Z., Hu N., Sun Z., Liu J. (2016). An Indoor Positioning Method for Smartphones Using Landmarks and PDR. Sensors.

[50] Kong T., Yao A., Chen Y., Sun F. HyperNet: Towards Accurate Region Proposal Generation and Joint Object Detection. Proceedings of the 2016 IEEE Conference on Computer Vision and Pattern Recognition (CVPR).

[51] Le-Hong P., Le A.-C. A Comparative Study of Neural Network Models for Sentence Classification. Proceedings of the 2018 5th NAFOSTED Conference on Information and Computer Science (NICS).

[52] Thakur D., Biswas S., Ho E., Chattopadhyay S. (2022). ConvAE-LSTM: Convolutional Autoencoder Long Short-Term Memory Network for Smartphone-Based Human Activity Recognition. IEEE Access.

[53] Wang L., Xu Y., Cheng J., Xia H., Yin J., Wu J. (2018). Human Action Recognition by Learning Spatio-Temporal Features with Deep Neural Networks. IEEE Access.

[54] Dua N., Singh S.N., Semwal V.B. (2021). Multi-Input CNN-GRU Based Human Activity Recognition Using Wearable Sensors. Computing.

[55] Ding X., Jiang T., Zhong Y., Huang Y., Li Z. (2021). Wi-Fi-Based Location-Independent Human Activity Recognition via Meta Learning. Sensors.

